# Association between Obstructive Sleep Apnea and Myocardial Infarction: A Systematic Review

**DOI:** 10.5935/abc.20170031

**Published:** 2017-04

**Authors:** Fernanda Porto, Yuri Saho Sakamoto, Cristina Salles

**Affiliations:** Escola Bahiana de Medicina e Saúde Pública, Salvador, BA - Brazil

**Keywords:** Sleep Apnea, Obstructive, Myocardial Infarction, Review, Adults, Polysomnography / methods, Sleep Wake Disorders

## Abstract

Obstructive sleep apnea (OSA) has been associated to cardiovascular risk factors. However, the association between OSA and cardiovascular disease is still controversial. The objective of the present study was to verify the association between OSA and myocardial infarction (MI). This is a systematic review of the literature performed through electronic data sources MEDLINE/PubMed, PubMed Central, Web of Science and BVS -*Biblioteca Virtual em Saúde* (Virtual Health Library). The descriptors used were: 'obstructive sleep apnea' AND 'polysomnography' AND 'myocardial infarction' AND 'adults NOT 'treatment.' The present work analysed three prospective studies, selected from 142 articles. The studies followed a total sample of 5,067 OSA patients, mostly composed by male participants. All patients underwent night polysomnography, and all studies found an association between OSA and fatal and non-fatal cardiovascular outcomes. Thus, we were able to observe that 644 (12.7%) of the 5,067 patients suffered MI or stroke, or required a revascularization procedure, and 25.6% of these cardiovascular events were fatal. MI was responsible for 29.5% of all 644 analysed outcomes. There is an association between OSA and MI, in male patients, and apnea and hypopnea index (AHI) are the most reliable markers.

## Introduction

Studies have demonstrated the association between obstructive sleep apnea (OSA) and myocardial infarction (MI).^1-5^ Up to 65% of patients who seek medical attention for a cardiovascular event are diagnosed with OSA.^1^ There is a need to study OSA's ability to predict cardiovascular events; some cohort studies, following apneic patients, have identified a high number of fatal and non-fatal cardiovascular outcomes.^2-8^ However, this association is still controversial.^1^ Results suggest that intermittent hypoxia could work as a protection factor for ischemic events, a phenomenon that has been observed in apneic individuals who develop a cardiac lesion that is less severe than those of patients with no OSA after a MI.^5^ Considering the prevalence of OSA, as well as the importance of cardiovascular diseases, the objective of the present systematic review of the literature was to verify the association between OSA and MI.

## Methods

### Study design and research strategy

This is a systematic review of literature and thus approval by a Research and Ethics Committee was not required. The search was performed in the electronic data sources MEDLINE/PubMed, PubMed Central® (PMC), Web of Science and BVS, through a combination of descriptors, including the terms of Medical Subject Heading (MeSH) and Health Sciences Descriptors (DeCS). The descriptors chosen to be used together were: "obstructive sleep apnea" AND "polysomnography" AND "myocardial infarction" AND "adults" NOT "treatment".

The search for descriptors on MEDLINE/PubMed yielded: *(((("obstructive sleep apnea"[All Fields]* OR *"sleep apnea, obstructive"[MeSH Terms]* OR *("sleep"[All Fields]* AND *"apnea"[All Fields]* AND *"obstructive"[All Fields]) OR "obstructive sleep apnea"[All Fields]* OR *("obstructive"[All Fields]* AND *"sleep"[All Fields]* AND *"apnea"[All Fields]))* AND *("polysomnography"[MeSH Terms] OR "polysomnography"[All Fields]))* AND *("myocardial infarction"[MeSH Terms]* OR *("myocardial"[All Fields]* AND *"infarction"[All Fields])* OR *"myocardial infarction"[All Fields]))* AND *("adult"[MeSH Terms] OR "adult"[All Fields]* OR *"adults"[All Fields]))*

After that, we did a manual search through selected articles.

### Inclusion and exclusion criteria

We included all cohort works found in the databases, with humans over 18 years of age, published in the last 10 years, in Portuguese, English and Spanish, with OSA diagnosis through polysomnography, with MI as one of the analysed outcomes. We excluded those in which 100% of the patients were under treatment for sleep disturbances (SD), all groups of treated apneic individuals, pregnant patients, those with other SDs, neurological or psychiatric diseases, and studies in which 100% of the population had previous coronary artery disease (CAD). We also excluded works whose population was approached in more than one study and that also had similar outcomes. In those cases, we considered the first work.

### Identification and selection of the studies

Two independent researchers read the titles and abstracts of each pre-selected work, separately identifying articles that met the inclusion and exclusion criteria. After this stage, each researcher read the complete articles that respected the criteria exposed in the abstract and selected only those compatible to the systematic review criteria. When there was doubt, a third researchers would be called in, but there was no disagreement between the first two researchers in this study.

### Data extraction

Two researchers were responsible for the data collection. Characteristics extracted from the studies were: title, authors, year of publication, science journal where it was published, publication medium, key-words, geographical origin, study design, sample size, supervision, financing, methods, research time, OSA diagnosis criterion, other results of the research, and conclusions. Moreover, participants' characteristics of each work were registered: number, gender, age, use of medication, comorbidities, number of patients who suffered a MI, and who received an OSA diagnosis, as well as the apnea and hypopnea index (AHI).

### Evaluation of methodological quality of selected articles

Two researchers read the articles, and each of them filled out a check list based on Strengthening the Reporting of Observational Studies in Epidemiology (STROBE).^9^ The selected articles were evaluated as having fulfilled each item completely or partially, or not at all. Articles considered as having acceptable quality were those that satisfactorily contemplated at least 11 aspects. In case of a disagreement between the two researchers, a third researcher would have been called in to assess the article, but that was not necessary. This systematic review also followed the recommendations of Preferred Reporting Items for Systematic Reviews and Meta-Analyses (PRISMA)^10^ and the step-by-step suggested by Cochrane Handbook,^11^ produced by The Cochrane Collaboration.

## Results

### Study identification and selection

**Figure 1:**
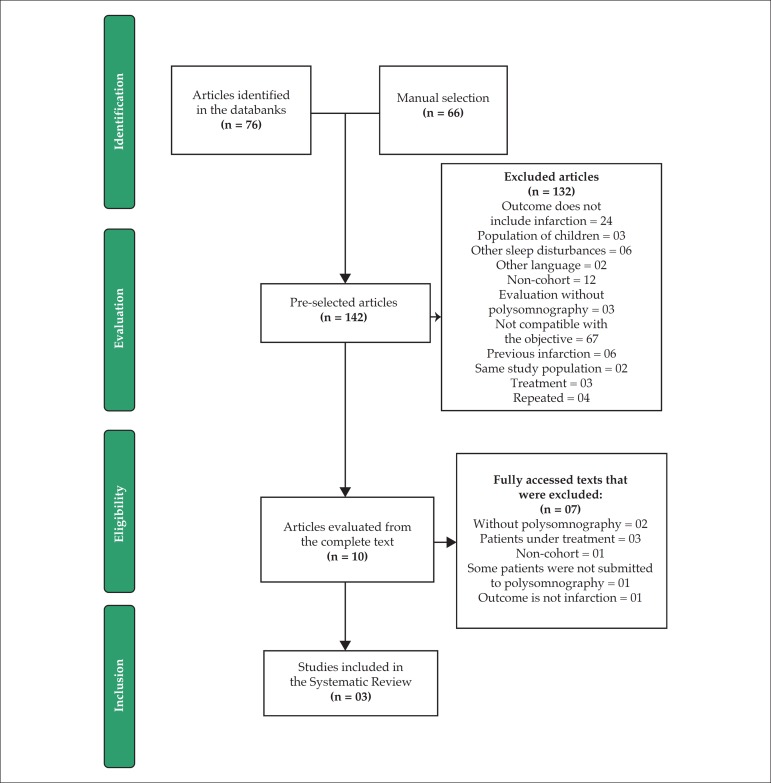
Flowchart of the study selection process.

The present systematic review gathered 76 articles through the search strategy outlined in electronic databases. Four of these articles were repeated in more than one source, as were two articles of the 66 found in the manual selection. Thus, from the 148 articles found, we counted 142 (Figure 1).

### Methodological evaluation of the studies

After complete reading of the selected works, we observed that all the articles satisfactorily fulfilled at least 16 aspects of the check-list.^9^ In the study by Buchner et al.^8^, 72.7% of the aspects were satisfactorily fulfilled; the one by Marin et al.^6^ were at 77.3%, and Gottlieb et al. reached the highest percentage (95.4%).

### Characteristics of the selected studies

The objective of the work by Gottlieb et al.,^6^ The Sleep Heart Health Study (SHHS), was to evaluate the relation between OSA, CAD incidence, and heart failure in a sample of the general community of men and women. The study included patients at 40 years of age or older, recruited among participants of population base studies about cardiovascular and pulmonary diseases, including Atherosclerosis Risk in Communities Study, Cardiovascular Health Study, Framingham Heart Study, Strong Heart Study, Tucson Health and Environment Study, and The New York University-Cornell Worksite and Hypertension Study. We excluded individuals whose polysomnography was inconclusive, those under treatment for OSA, with low quality data, previous CAD or heart failure, without follow-up data, or incomplete data on body mass index (BMI), smoking, blood pressure and use of medications that refused to participate in the study.

SHHS^6^ analyzed a total sample of 4,422 patients, including 2,434 who had AHI < 5. We also observed that 5.5% of 3,794 patients were diagnosed with OSA five years after the beginning of the study, and of those, 2.1% referred treatment for OSA, but were excluded without significantly altering the results. In this study, 43.3% of the population was male. Among the patients from the sample AHI ≥ 5, the male population represented 55.23% of the sample.

The objective proposed by Marin et al.^7^ was to compare the incidence of fatal and non-fatal cardiovascular events in simple snorers, untreated OSA patients, patients with continuous positive airway pressure (CPAP) and healthy men recruited among the general population. In this study, the sample included only men with OSA or simple snoring from sleep clinics, and a population base sample of healthy men matched by age and BMI with severe apneic patients, between January 1992 and December 1994. The healthy men were recruited from the database of Zaragoza Sleep Apnea Study. 

Buchner et al.^8^ prospectively investigated cardiovascular outcomes in treated versus non-treated OSA patients. For this study, recruitment included all patients with suspected sleep-related obstructive respiratory disturbances, admitted in a sleep clinic through non-selected referral by primary or secondary care physicians between 1993 and 1998. Snorers without apnea and patients with central sleep apnea, Cheyne-Stokes breathing, hypoventilation syndromes or periodic limb movement during sleep were excluded from the analysis. The patient sample in this study was predominantly male - 85.5% of a total of 449, and 83.5% were untreated apneic individuals.

Mean follow-up among the three studies was 8.2 ± 0.99 years. The number of patients varied between 449 and 4,422. Only Gottlieb et al.^6^ evaluated OSA impact on the appearance of CAD and, thus, none of the participants received OSA treatment.

Comorbidities and risk factors common to the three studies were systemic arterial hypertension (SAH), diabetes mellitus (DM), dyslipidemia, and smoking. However, only DM and smoking were related in the same way by all three studies (number of DM patients and smokers). Treatment with insulin or oral antidiabetic drugs was mentioned only by Buchner et al.^8^ Regarding SAH and dyslipidemia, Gottlieb et al.^6^ reported only the number of patients on antihypertensives or lipid-lowering drugs, and Marin et al.^7^ registered only the total number of SAH and dyslipidemia patients. On the other hand, Buchner et al.^8^ reported the initial number of SAH patients from those who started follow-up with antihypertensives, and the number of patients, at the end of follow-up, under treatment for this pathology; the same happened for cases of dyslipidemia.

The sample of 5,067 patients analyzed in this systematic review must be considered in their isolated groups, considering there are important differences between the participants of each study regarding comorbidities. This situation must be illustrated with proportions, in the studies by Gottlieb et al.,^6^ Marin et al.,^7^ and Buchner et al.,^8^ for the number of patients with AHI ≥ 5 and SAH or, in the case of SHHA,^6^ the use of antihypertensives (39.2% vs 28.5% vs 69.4%)/ dyslipidemia or, in the case of SHHS,^6^ the use of hypolipidemic drugs (7.5% vs 10.1% vs 57.6%). Gottlieb et al.^6^ reported the percentage of patients who were on antihypertensives and hypolipidemic drugs, and, therefore, patients with untreated dyslipidemia or SAH were not registered. Marin et al.^7^ did not report the number of patients under treatment (Table 1).

**Table 1:** General characteristics of selected studies

Authors	Country Year	Journal	N initial	N AHI < 5	N AHI > 5	NTreated apneic	NNon-treated apneic	*Follow-up*Mean in years	% Male	Reported comorbidities
Gottlieb et al.^6^	USA. 2010	Circulation	4.422	2.434	1.988	79	1.988	8.7	43.5%	SAH. DM. Dyslipidemia. Smoking
Marin et al.^7^	Spain. 2005	The Lancet	1.651	264	1.010	372	638	10	100%	SAH. DM. Dyslipidemia. Cardiovascular Disease. Smoking. Alcoholism
Buchner et al.^8^	Germany 2007	American Journal of Respiratory and Critical care Medicine	449	0	449	364	85	6	85.5%	SAH. DM. Dyslipidemia. Coronary Disease. Peripheral Artery Disease. STROKE. Neoplastic Disease. COPD and Smoking

OSA: obstructive sleep apnea; AHI: apnea and hypopnea index; SAH: systemic arterial hypertension; DM: diabetes mellitus; COPD: chronic obstructive pulmonary disease.

Clinical characteristics that compose the profile of groups common to all three studies are: participants' ages, BMI, number of DM patients, number of smokers and AHI, but no statistically significant conclusion was drawn on smoking (Tables 2-4).

**Table 2:** Age of patients from studied samples according to apnea and hypopnea index

Authors	Median age (interquartile range); Mean ± Standard Deviation					p
	AHI < 5		AHI ≥ 5			
	< 5		5 a < 15	≥ 15 a ≤ 30	>30	
Gottlieb et al.^6^	Men	61(54.7)	64(57.7)	64(57.7)	65(58.7)	NI
	Women	60(50.7)	66(58.7)	66(58.7)	65(58.7)	NI
Marin et al.^7^	49.6 ± 8.1		50.3 ± 8.1		49.9 ± 7.2	NI
Buchner et al.^8^	NA		57.8 ± 10.2			NS

NS: non-significant; NI: not informed; NA: non-applicable; AHI: apnea and hypopnea index.

**Table 4:** Number of patients with diabetes mellitus in the studied samples according to apnea and hypopnea index

Authors	Diabetes per group N(%)					p
	AHI < 5		AHI ≥ 5			
		< 5	5 a < 15	≥ 15 a ≤ 30	> 30	
Gottlieb et al.^6^	Men	73 (8.8)	77(12.0)	39(13.8)	29(16.9)	NI
	Women	123 (7.7)	82 (13.4)	33 (16.8)	14 (16.7)	NI
Marin et al.^7^	(6.1)		(8.5)		(9.9)	NI
Buchner et al.^8^	NA		13 (15.2)			NS

NS: non-significant; NI: not informed; NA: non-applicable; AHI: apnea and hypopnea index.

### Incidence of myocardial infarction

Gottlieb et al.^6^ evaluated 4,433 individuals, of which 473 CAD cases were recorded - 76 deaths from CAD, 185 MI, 212 revascularization procedures - with an incidence of 20.1 events per 1000 person-years among men, while in women, this rate was 8.7 events per 1000 person-years. These data showed the increase, in men, of the incidence rate of revascularization according to the severity of OSA, whereas, in women, these values were less evident.

In the population of 403 men with mild to moderate OSA from the study by Marin et al.,^7^ by associating the event rate to the severity of OSA, they observed 36 non-fatal cardiac events, with the incidence rate of events at 8.9 events per 1000 person-years, as well as 22 deaths from cardiovascular causes with a rate of 5.5 events per 1000 person-years. Among the 235 men with severe OSA, 50 non-fatal cardiovascular events were registered with an incidence rate of 21.3 events per 1000 person-years, and 25 deaths from cardiovascular causes at a rate of 10.6 events per 1000 person-years. This study did not differentiate the equivalent data of the different outcomes: fatal or non-fatal MI, fatal or non-fatal stroke, and acute coronary insufficiency requiring revascularization surgery or percutaneous transluminal coronary angiography, or both.

Buchner et al.,^8^ evaluating 85 patients, concluded that 28.3% had the following outcome: five MIs, 25 revascularization procedures, five strokes, and three deaths from cardiovascular causes. Of these patients, 20 (23.5%) were diagnosed with mild-moderate OSA.^8^

## Discussion

The present systematic literature review analysed three prospective works that followed a total sample of 5,067 patients, between men and women, of which 53.5% had different degrees of untreated OSA diagnosed by polysomnography. All the studies found an association between OSA and fatal and non-fatal cardiovascular outcomes. It was observed that 644 (12.7%) of the 5,067 patients suffered MI or stroke or required a revascularization procedure, and 25.6% of these events were fatal. This is a relevant number considering the main cause of death and disability, in Brazil and in the world, is constituted by cardiovascular diseases.^12^

According to the American Heart Association^13^ (AHA), one in every seven deaths in the USA are caused by cardiac diseases - every 34 seconds, an American has a coronary event, and every minute and 24 seconds, there is a death from MI.^13^ Thus, the data of the present study are in agreement with the current literature, since 190 MI cases were counted in the analyzed group (3.75%). However, this final number may be even higher, since we did not consider the percentage of the group studied by Marin et al.,^7^ because there was no information on how many patients had MIs. These authors only reported that the type and frequency of the different outcomes did not differ between the studied groups.^7^

Some studies demonstrated an association between MI and OSA. ^2,4,14-18^ Shah et al.^4^ concluded that OSA increases the risk of MI, revascularization procedures and cardiovascular death, regardless of risk factors, such as SAH, in patients over 50 years of age. However, this cohort did not exclude patients who received treatment for OSA during 2.9 years of follow-up.^4^ With base on evidence that treatment with CPAP decreases the risk of fatal and non-fatal cardiovascular events, ^1,14,19^ Shah et al.^4^ said that the study design did not allow them to work with adhesion to treatment and/or treatment effects, and such finding would polarize the results to zero. Nevertheless, in this study, 86 patients (6.1%) had some of the outcomes - 74 had OSA and, of those, 21 had MI, as well as 33 cases registered as cardiovascular death.^4^

In contrast, Kendzerska et al.,^2^ in a study whose objective was to determine if OSA independently increases the risk of coronary events, concluded that AHI was associated to composite cardiovascular outcome in a univariate analysis, but not in a multivariate analysis. The argument used to explain this finding was that, possibly, studies with large community bases may not include important predictors related to OSA, or may selectively relate subgroup analyses conclusions. Kendzerska et al.^2^ considered the history referred by the patients, such as smoking, MI, myocardial revascularization surgery, stroke, SAH and/or pulmonary disease. With the justification that recovery by CPAP was not associated to the risk of an event, the patients who needed treatment were not excluded, and in the analysis of the untreated patients, in relation to the complete sample, all predictors remained significantly associated to the outcome, except for daytime drowsiness.^2^

Regarding the inclusion of cardiovascular risk factors, the only comorbidities equally studied by the authors of the works, in this systematic review, were DM and smoking. Age, BMI, and AHI were also mentioned by all the authors. Only Gottlieb et al.^6^ excluded, at the beginning of the study, patients with previous CAD or heart failure, while the other two studies^7,8^ included and registered these cases. Several factors, including the strict relation between obesity and OSA, make it difficult to understand the effect of each pathology and the synergy between them.^14^ Moreover, multiple comorbidities are present in OSA patients with metabolic syndrome, DM, and cardiovascular disease itself - a situation that creates the challenge of explaining if secondary abnormalities are caused by OSA or other pre-existing conditions.^14^

Kendzerska et al.^2^ and Shah et al.^4^ also included potential factors of confusion in their works. What can be observed, in fact, is that there are several cardiovascular risk factors that are seldom seen together in only one study, including family history - a target topic for the study by Gami et al.^15^ These authors performed a cross-section study with over 500 apneic individuals, diagnosed by polysomnography, and found a strong and independent association between OSA and family history of premature death from cardiovascular disease.^15^ This association shows important implications for the understanding of cardiovascular risk in these patients and raises this hypothesis so that future cohort works can be performed.^15^

In the current literature, the association between OSA and MI is shown by the proportion of events that occur throughout the years. Gottlieb et al.^6^ reported that the association shown by them is considerably weaker that that of previous studies. The authors demonstrated the curves for the rate of survival free of coronary disease and heart failure, highlighting a drop in these rates throughout the years, according to the severity of the OSA. This weak association can be attributed to three main aspects: studies that work with cerebrovascular diseases, together with cardiovascular diseases, have higher rates of outcomes; studies that overestimate untreated patients such as those who refuse treatment, and thus neglect other health issues; and the study by Gottlieb et al.^6^ that selected a sample from a community that did not seek sleep medicine services and, therefore, did not present signs or symptoms of OSA, with no clinical profile of these participants having been registered. It is possible that OSA, in such individuals, may bring a cardiovascular risk that is inferior to that of individuals who go to a sleep clinic for suspected OSA.^6^

Indeed, there is evidence that, in OSA patients without daytime drowsiness, treatment with CPAP does not offer a significant reduction in the incidence of SAH or cardiovascular events, though Barbe et al.^20^ have admitted low power to detect differences between the groups with or without complaints. Regarding cardiovascular events incidence rates, Marin et al.^7^ registered but did not distinguish gender and separated values by OSA severity degree and fatal or non-fatal outcome. On the other hand, Buchner et al.^8^ did not record this information.

Free survival rate was also not checked in the study by Marin et al.,^7^ whereas Buchner et al.^8^ estimated a survival free of disease in patients with mild to moderate OSA without pre-existing cardiovascular disease after 10 years at 90.7% in groups of treated patients, and at 68.5% in non-treated patients groups.

AHI translates the frequency of apneas and hypopneas per hour of sleep and works as a measurement of OSA severity often related to advanced age, male gender, obesity, daytime drowsiness, and the presence of comorbidities.^14^ Regarding this variable, it was observed that, in the study by Marin et al.,^7^ mean value of AHI in patients with mild to moderate OSA was 18.2 ev/h and in those with severe apnea it was 43.3 ev/h. In the sample of untreated patients from the study by Buchner's et al.^8^, mean value of AHI for all OSA patients groups was 15.3% ev/h, compatible with the frequency of mild, moderate, and severe apneic individuals: 56.7%, 28.2%, and 7.1%, respectively. In the group from Gottlieb et al.,^6^ AHI median was 6.2 ev/h (men) and 2.7 ev/h (women), apparently including the values of patients with AHI < 5. This inclusion of 829 healthy men and 1605 healthy women may have interfered in AHI values of SHHA.^6^ The authors also observed that the association of AHI with heart failure and CAD occurred in patients with AHI ≥ 30.

The patient sample, in this systematic review, was mostly composed by adults over 40 years of age, and this was also the inclusion criteria used by Gottlieb et al.^6^ In the study by Marin et al,^7^ the mean age of severe apneic patients was 49.9 years, the lowest mean registered among the groups; Buchner et al.^8^ reported a mean age of 57.8 years among all untreated apneic patients. It is important to keep those age values in mind, because He et al.^21^ suggested that OSA can have more severe cardiovascular consequences in individuals under 50 years of age. Studies have also demonstrated that younger individuals, with OSA, can be more prone to SAH,^22^ atrial fibrillation,^23^ and have a higher risk of death for any other cause.^24^ With this evidence, it is necessary to understand if an aggressive therapeutic and diagnostic strategy would benefit younger and middle-aged individuals with OSA.^14^ For that, other characteristics must be considered in future studies, such as ethnicity, gender and other demographic data.^14^

The relative consideration to the age factor allowed Gottlieb et al.^6^ to show that cardiovascular risk associated to OSA decreases with age.^25^ The SHHS^6^ cohort, whose mean age was 62 years, may have underestimated the true cardiovascular risk in these patients. The authors of SHHS^6^ argued that cardiovascular risk can decrease with age due to biological differences in OSA's pathophysiology between patients of different ages. The authors reported that the "healthy survivor" effect is a probable cause for a bias towards a null result, since apneic individuals, more susceptible to OSA's cardiovascular effects, are also more prone to cardiovascular diseases and have a higher risk of death than those with OSA who are resistant to cardiovascular consequences.^6^

In this work, 51.23% of the 5,067 analysed patients were male - Marin et al.^7^ included only men in their study. By only observing the population of patients with AHI ≥ 5, we can see that the percentage increases to 65.57%. After statistical analysis and matching for age, ethnicity, smoking, and BMI, there was a strong association of AHI with heart failure in men, but not in women, according to Gottlieb et al.^6^ In the same way, event rates increased with the severity of OSA in men, but that was not demonstrated in women.^6^

Buchner et al.^8^ had only 16.5% of women in the non-treated group, and stated they could not extrapolate their results to this population. Considering the abovementioned facts, the present work will also restrict itself to the analysis of OSA in the male population - in the general population, the ratio between men and women with OSA is estimated at 2:1 to 3:1.^8^

Regarding factors of confusion, such as SAH, DM, dyslipidemia, we can observe that treatment of these pathologies has a relevant impact on outcomes such as MI.^26-28^ They are part of the metabolic syndrome, which represents an important risk factor for CAD,^26-28^ and it is important to know the therapeutic status of the population. Gottlieb et al.^6^ worked only with patients under treatment, an aspect that may have interfered in the weak association found between OSA and MI. Buchner et al.^8^ also registered the number of patients under treatment, and it was also the study with the highest number of comorbidities, an aspect that may have interfered in the higher number of non-fatal cardiovascular events, revascularization procedures, and infarctions observed in the three articles. Marin et al.,^7^ in turn, did not report the therapeutic status of the population, and it was also the only study that registered a large number of fatal cardiovascular events.

Studies show the prevalence of DM type II in the population of apneic individuals.^29-31^ Catecholamine elevation together with sleep deprivation^32^ are associated to insulin resistance. There are also data that suggest an association between OSA and glucose intolerance regardless of BMI.^33,34^ Chen et al.^35^ concluded, in a meta-analysis, that treatment with CPAP, even though it does not alter glycated hemoglobin levels, it significantly improves insulin resistance, positively impacting on DM symptoms. In the present systematic review, 12.7% of the 2,711 patients with AHI ≥ 5 also had DM, whereas Gottlieb et al.,^6^ Marin et al.,^7^ and Buchner et al.^8^ registered 13.7% vs 9% vs 15.2%, respectively.

Regarding BMI, the highest median in the study by Gottlieb et al.^6^ was 31.1 kg/m^2^, in apneic men with AHI ≥ 30 ev/h. Marin et al.^7^ registered a mean of 30.3 kg/m^2^ in the untreated severe OSA group, and 27.5 kg/m^2^ for mild to moderate OSA. Buchner et al.^8^ reported a mean of 29.3 kg/m^2^ for the entire sample, 55% of which had mild apnea. Resta et al.^36^ and Newman et al.^37^ observed a higher frequency of OSA among obese individuals, and, similarly, Silva et al.^38^ concluded that obesity is a determining factor in OSA. Newman et al.^37^ estimated that obese individuals can have approximately two times more chances of developing OSA. An elevated BMI is also associated to an increase in mortality from several chronic pathologies, especially cardiovascular diseases.^39^ Framingham demonstrated that an elevated weight increases the risk of CAD, regardless of other risk factors.^40^ These observations help us understand the importance of the high BMI value registered by the articles, in this systematic review, as well as the direct relation between BMI and OSA severity. It is also important to know the personal history of cardiovascular disease of the individuals in the analyzed sample in this systematic review. Gottlieb et al.,^6^ Marin et al.,^7^ and Buchner et al.^8^ registered 0% vs 6.3% vs 70.5%, respectively.

From the exposed factors, we can observe that Buchner et al.^8^ gathered conditions that favored cardiovascular outcomes: SAH, DM, dyslipidemia, as well as the high percentage of patients with a history of cardiovascular disease not being simple independent confusion factors of OSA. These factors can contribute to the adverse effects of OSA in cardiovascular outcomes; therefore, the higher proportion of cardiovascular risk factors and diseases could explain the number of cases and treatment benefits in patients with mild to moderate OSA, as reported by the authors.

The understanding of OSA effects may suggest explanations for the association of this pathology with MI. The prevalence of SDs in CAD patients is up to two times higher than in individuals without CAD. Bhama et al.^41^ reported a prevalence of up to 30% of apneic individuals among patients with CAD.

There are pathophysiological mechanisms that suggest the contribution of OSA in the origin and progression of CAD: severe intermittent hypoxemia, acidosis, increase of blood pressure and sympathetic vasoconstriction, together with simultaneous changes in transmural, intrathoracic and cardiac pressures.^14^ These factors strengthen the argument that OSA has a strong potential to trigger cardiac ischemia.^14^ In the long run, the mechanisms of cardiac and vascular diseases, including endothelial dysfunction and systemic inflammation, can damage the structures of coronary arteries.^14^ Sorajja et al.,^42^ when studying patients with no history of CAD, observed the presence of an important calcification in the coronary arteries of OSA patients, through the calcification score = 9 (Agatston units) and zero score in patients without OSA (p < 0.001).

There are reports that the reduced number of cardiovascular events separated amongst themselves, and the variation of definitions used by research limit the conclusions of studies that address this theme.^18^ The present systematic review also had as limitations the heterogeneity of the selected studies in relation to aspects such as objective, clinical and polysomnographic profile of the patients, as well as the difference in the presentation of patient groups and their classification regarding AHI.

This context demonstrated the challenges of investigating the causal relation between OSA and CAD, considering both conditions are chronic and have long latency periods before the appearance of complaints.^14^ Moreover, both pathologies also have multifactorial origins with an overlap of common risk factors such as gender, age, obesity, and smoking.^14,43^

Defining the causal relationship between CAD and OSA means clarifying the necessary care apneic individuals must have regarding the prevention of MI. Making sleep apnea a marker for cardiac disease implies early tracking of these patients, as well as an incentive to treat this disease that is associated to numerous cardiovascular consequences. Endothelial, neuro-hormonal and metabolic alterations cannot be overlooked, even if it seems complex to dissociate the onset of CAD and OSA, because it is only thus that we can understand if OSA can interfere in the development or aggravation of CAD. OSA can be treated and, therefore, if this is confirmed, it can be a controllable determinant of CAD.

## Conclusion

This systematic review has shown that there is an association between OSA and MI. We were able to observe that this association was higher among men, and that AHI was considered one of the markers for this relationship.

## Figures and Tables

**Table 3:** Body mass index of patients from studied samples according to apnea and hypopnea index

Authors	BMI Kg/m^2^ Median (interquartile range) ; Mean ± Standard Deviation					p
	AHI < 5		AHI ≥ 5			
		< 5	5 a < 15	≥ 15 a ≤ 30	> 30	
Gottlieb et al.^6^	Men	27.0(24.6. 29.3)	28.8(26.2. 31.4)	29.7(26.9. 33.5)	31.3(27.9. 34.9)	NI
	Women	26.3 (23.6. 29.8)	29.9(26.1. 34.1)	32.5(27.3. 36.9)	34.3(29.1.39.6)	NI
Marin et al.^7^	29.8 ± 4.4		27.5 ± 4.4*		30.3 ± 4.2	< 0.0001*
Buchner et al.^8^	NA		29.3 ± 5.4			0.003

NS: non-significant; NI: not informed; NA: non-applicable; AHI: apnea and hypopnea index; BMI: body mass index;

(*) p < 0.0001 vs men with AHI < 5.
